# Chemotherapy alone or combined chemotherapy and involved field radiotherapy in favorable risk early-stage classical Hodgkin lymphoma-a 10 years experience

**DOI:** 10.12669/pjms.326.11080

**Published:** 2016

**Authors:** Sheeraz Ali, Abdul Basit, Ather S. Kazmi, Armughan Sidhu, Farhana Badar, Abdul Hameed

**Affiliations:** 1 Dr. Sheeraz Ali, MBBS, FCPS (Medicine), Fellow Medical Oncology, Department of Medical Oncology, Shaukat Khanum Memorial Cancer Hospital & Research Centre, Lahore, Pakistan; 2 Dr. Abdul Basit, MBBS, FCPS (Medicine), Fellow Medical Oncology, Department of Medical Oncology, Shaukat Khanum Memorial Cancer Hospital & Research Centre, Lahore, Pakistan; 3 Dr. Ather Saeed Kazmi, MBBS, MRCP FRCR. Clinical Oncologist, Department of Medical Oncology, Shaukat Khanum Memorial Cancer Hospital & Research Centre, Lahore, Pakistan; 4 Dr. Farhana Badar, Sr. Biostatistician & Cancer Epidemiologist Cancer Registry, & Clinical Data Management, Department of Cancer Registry and Clinical Data Management, Shaukat Khanum Memorial Cancer Hospital & Research Centre, Lahore, Pakistan; 5 Dr. Armughan Sidhu, MBBS. Resident Medical Oncology, Department of Medical Oncology, Shaukat Khanum Memorial Cancer Hospital & Research Centre, Lahore, Pakistan; 6 Dr. Abdul Hameed MBBS, MD, FRCP (Edin). Consultant Hematologist, Department of Medical Oncology, Shaukat Khanum Memorial Cancer Hospital & Research Centre, Lahore, Pakistan

**Keywords:** Hodgkin Disease, Survival Analysis, Combined Modality Therapy, Antineoplastic Combined Chemotherapy Protocols, Radiotherapy

## Abstract

**Objective:**

To determine the outcome of patients with early-stage (stage I-II) favorable risk classical Hodgkin lymphoma treated with chemotherapy alone or combined modality treatment (CMT) utilizing chemotherapy and involved field radiotherapy.

**Methods:**

This retrospective study was done at Department of Medical oncology, Shaukat Khanum Memorial Cancer Hospital & Research Centre, Lahore, Pakistan from January 2004 to December 2013.

**Results:**

There were 101 patients, with male predominance (71.3%). Mean age was 34 years. Sixty three (62.4%) patients received CMT and 38 (37.6%) patients had chemotherapy alone. Ninety eight percent patients had ABVD chemotherapy. Dose of radiotherapy ranged from 20 to 36 gray. Difference between baseline characteristics and major toxicities among the two groups was insignificant. Patients treated with CMT had better overall survival compared to chemotherapy alone: 100% versus 91% at five years and 96% versus 81% at 10 years, respectively (p=0.03). Progression free survival was also better with CMT against chemotherapy alone at five years (98% versus 81%) and 10 years (82% versus 71%) (p=0.01).

**Conclusion:**

Favorable risk classical Hodgkin lymphoma patients had better overall survival and progression free survival when treated with CMT against chemotherapy alone

## INTRODUCTION

Over the past 2-3 decades, incidence of Hodgkin lymphoma (HL) in the western world has remained fairly steady at about 3 per 100000 which are considerably lower than that of non-Hodgkin lymphoma. Reported incidence from Asian countries is somewhat less than this.^1^ Hodgkin lymphoma is divided in Nodular lymphocyte predominant Hodgkin lymphoma and Classical Hodgkin lymphoma which is further sub-classified into four histological types, nodular sclerosis, mixed cellularity, lymphocyte depleted and lymphocyte rich.^2^

Advanced age, mediastinal bulk disease, raised ESR, Bsymptoms (fever > 38°C, drenching night sweats, unexplained weight loss >10% of total body weight over 6 months) and number of involved nodal sites are all known adverse prognostic factors.^3^

These risk factors are usedto segregate patients with early-stage Hodgkin lymphoma (Ann Arbor stage I and II) into 2 groups i.e., favorable-absence of aforementioned factors and unfavorable-presence of these factors.^4^,^5^

Subtotal nodal irradiation (STNI) alone, which consisted of sequential irradiation of the cervical, axillary, mediastinal, and hilarlymphnodes followed by spleen (if present) and para-aortic nodes; was standard treatment in the past and it achieved relapse free survival of 80% at ten years.^6^,^7^ This approach resulted in potentially fatal long term complications like secondary leukemia and solid organ tumors, infertility, cardiovascular and pulmonarydisease.^8^ Efforts were made to reduce long term toxicities by reduction of radiation dose and area of exposure-involved field rather than extended filed.^5^,^9^ Some experts even recommend involved site radiotherapy in an effort to minimize side effects without compromising the outcome.^10^ Combined modality treatment (CMT) including chemotherapy and involved field radiation showed better results and with low toxicity profile.^11^

Long term safety concerns which emerged with the use of radiotherapy led to use of chemotherapy alone for treatment of early-stage favorable risk HL resulting in more or less similar outcome as shown with CMT.^12^,^13^ Recent trials for patients with limited-stage HL have demonstrated that treatment with chemotherapy plus involved-field radiation therapy (IFRT) and chemotherapy alone with Doxorubicin (Adriamycin), Bleomycin, Vinblastine, and Dacarbazine (ABVD) may both be acceptable options for these patients.^5^,^14^ Some studies have highlighted the role of interim PET/CT to guide treatment; however, this approach may not be applicable everywhere because the availability of this imaging modality is still sparse in many countries.^15^,^16^

Most optimal treatment regimen for favorable risk early-stage HL remains unknown; however, many experts are in the favor of CMT for early stage Hodgkin’s lymphoma.^17^,^18^ The aim of this study was to determine the outcomes of our patients who presented with favorable risk early-stage Hodgkin lymphoma who were treated either with chemotherapy alone or chemotherapy plus IFRT.

## METHODS

From January 2004 to December 2013, newly diagnosed patients of early-stage (stage I & II) classical HL with favorable risk prognostic features were included in this retrospective analysis. All patients were treated in our institution and this analysis was performed after approval from institutional review board. Favorable risk group was defined according to National Comprehensive Cancer Network (NCCN) guidelines as having none of the unfavorable prognostic markers like B symptoms, ESR more than 50, bulky disease and more than three nodal sites involvements as per Ann Arbor staging system.^18^ Bulky disease was defined as single node or nodal mass of more than 10 cm or a mediastinal mass more than 1/3 in width of internal transverse diameter of thorax on chest X ray PA view at the level of interspace between T5 and T6vertebrae.^19^

Characteristics of patients at presentation like Ann Arbor stage, histological type, sex, age, presence or absence of bulky disease, number of involved sites, ESR, extra nodal site involvement, presence or absence of B symptoms were noted. Number of cycles, type of chemotherapy, dose and field of radiation given were also recorded. Any evidence suggestive of significant acute or long term toxicity was also documented.

Patients were divided in two groups based on whether they were treated with chemotherapy alone or combined modality treatment (CMT) comprising of chemotherapy followed by radiotherapy. Baseline characteristics in both groups were compared using cross tab and chi square test. Number of patients requiring inpatient admission due to therapy related complications or other significant toxicities were also compared using cross tab and chi square test. Progression free and overall survival for both groups of patients was calculated using Kaplan Meier method and log rank test was used for comparison.

We took the difference between the date of registration for treatment at the hospital and the date of final outcome-death or last follow-up, as overall survival. In order to perform time to event analyses, we defined death as the event while patients who were alive at last follow-up or were lost to follow-up were considered censored. Progression free survival was calculated as time period from date of registration to the time of first event (i.e., relapse, progression or death). Patients who were on follow-up without relapse or progression and those who lost to follow-up before final analysis were censored.

## RESULTS

Total number of patients was 101. Mean age of patients was 34 years (range 18-75 years). Seventy two (71.3%) patients were male and 29(28.7%) were female. Patients were equally distributed according to stage: 46.5% stage IA versus 53.5% stage IIA disease. Baseline characteristics and comparison of patients according to mode of treatment is summarized in Table-I. The distribution of patients in two groups was not significantly different according to these characteristics. Only two patients out of 101 had extra nodal site involvement. Sixty three (62.4%) patients received chemotherapy along with involved field radiotherapy and 38 (37.6%) patients had chemotherapy alone. All patients received ABVD (Adriamycin, Bleomycin, Vinblastine and Dacarbazine) chemotherapy regimen except 2: One of them received VEPEMB regimen and other patient received ChlVPP chemotherapy.

**Table-I:** Baseline characteristics of patients in each group along with comparison.

Characteristics	All patient(n=101) No.(%) of patients	Combined modality* (n=63) No.(%) of patients	Chemotherapy(n=38) No.(%) of patients	p value
Sex				0.34
Male	72(71.3)	47(74.6)	25(65.8)	
Female	29(28.7)	16(25.4)	13(34.2)	
Age				0.12
< 40 years	76(75.2)	45(71.4)	31(81.6)	
> 40 years	25(24.8)	18(28.6)	7(18.4)	
Stage				0.24
I A	47(46.5)	33(52.4)	14(36.8)	
II A	54(53.5)	30(47.6)	24(63.2)	
Histology				0.16
Mixed cellularity	64(63.4)	36(57.1)	28(73.7)	
Nodular sclerosis	29(28.7)	21(33.4)	8(21)	
Others†	8(7.9)	6(9.5)	2(5.3)	
Baseline ESR				0.25
< 25	68(67.3)	45(71.4)	23(60.5)	
> 25	33(32.7)	18(28.6)	15(39.5)	
Number of Involved Nodal Sites				0.25
1	47(46.5)	33(52.4)	14(36.8)	
2	45(44.5)	25(39.7)	20(52.7)	
3	9(9)	5(7.9)	4(10.5)	

* Combined modality consists of chemotherapy and involved field radiotherapy.

† Others include lymphocyte depleted and lymphocyte rich histology of Classic Hodgkin’s lymphoma.

Median follow up time was 56.7 months. Patients treated with chemotherapy alone had on average 5.2±1.05 chemotherapy cycles versus 3.6 ± 1.18 cycles among patients treated with chemotherapy in combination with involve field radiotherapy (IFRT). Dose of radiotherapy given was 20 to 36Gray. All patients had involved field radiotherapy.

There were 5 death documented, one in combined modality group and four in chemotherapy alone group. One patient in chemotherapy alone group died of fulminant hepatic failure due hepatitis B during the course of treatment. Seven patients relapsed, two in combined modality group and five in chemotherapy alone group. All of them had multiple sites involvement at relapse. Toxicity profile did not showed any significant difference among treatment groups. Number of admission due to therapy related complications and other significant side effects are summarized in Table-II.

**Table-II:** Major adverse events associated with therapy in each group along with comparison.

Adverse Events	Combined modality* (n=63) No.(%) of patients	Chemotherapy(n=38) No.(%) of patients	p value
Admissions (Therapy related complications)	4(6.3)	3(7.9)	0.27
Bleomycin toxicity	2(3.2)	1(2.6)	0.87
Cardiotoxicity	1(1.6)	0(0)	0.43
Hypothyroidism	2(3.2)	0(0)	0.26
Secondary malignancy	1(1.6)	0(0)	0.43

* Combined modality consists of chemotherapy and involved field radiotherapy.

**Fig.1:**
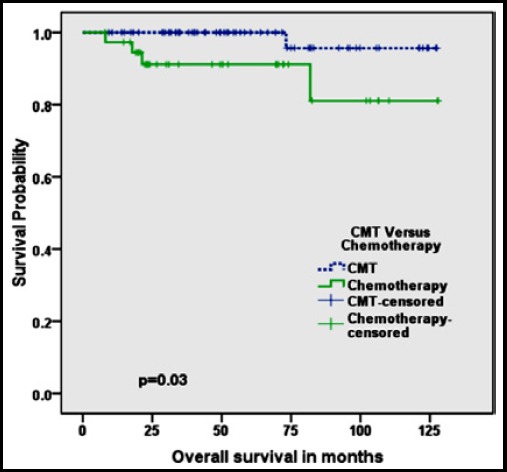
Kaplan Meier Curve showing Overall Survival of combined modality treatment (CMT) versus Chemotherapy alone.

**Fig.2:**
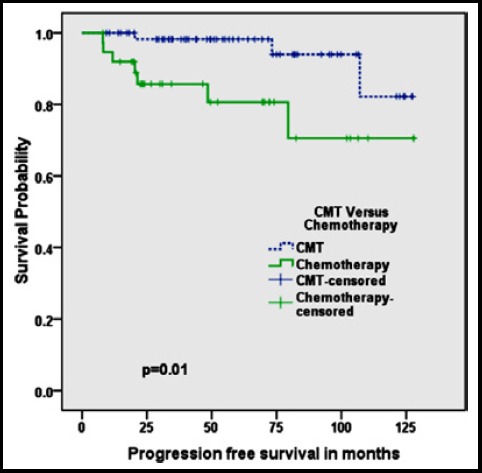
Kaplan Meier Curve showing Progression Free Survival of combined modality treatment (CMT) versus Chemotherapy alone.

Patients treated with CMT had significantly better overall survival: five year survival of 100% and 10 years survival of 96% compared to five years survival of 91% and ten years survival of 81% for patients treated with chemotherapy alone, respectively(p=0.03) (Fig.1). Similarly progression free survival for patients treated with CMT was 98% at 5 years and 82% at 10 years which was better than patients treated with chemotherapy alone showing 5 years survival of 81% and 10 years survival of 70%(p=0.01)(Fig.2).

## DISCUSSION

Hodgkin lymphomais a curable malignancy and this fact was pointed out many decades ago. Since then different treatment approaches have been used to increase survival with minimal toxicities. Radiation was the first treatment modality leading to cure and remained standard of care for quite some time.^20^ However, long-term follow-up revealed serious side effects including second malignancies, organ damages such as cardiac, pulmonary and gonadal toxicities.^8^,^21^ In particular, risk of breast cancer increased significantly in women who had thoracic radiation.^22^ During the early years, the radiation field used to be extended with higher doses which increased the risk of these complications.^20^,^21^ Chemotherapeutic agents such as chlormethamine was introduced in middle of previous century which resulted in better disease control but was also associated with significant toxicity profile with regard to second hematologic malignancies.^23^ In HL, early deaths due to disease usually occur within five years. Late fatalities are mostly due to complications of therapy (second cancer/organ damage, etc.).^24^ Addition of ABVD regimen resulted in excellent outcomes with lesser side effects. Different trials have compared ABVD with more intensive regimens with different results with regard to OS however, intensive regimens such as BEACOPP was associated with more side effects.^25^,^26^ To date, ABVD is the most used first line therapy for HL.^27^ Role of radiotherapy in the treatment of early stage HL still remains significant. Recent studies on the treatment of early stage HL with combination of ABVD and radiotherapy had been very encouraging.^5^,^11^

In our study, we looked at the outcomes of the early stage (I/II) favorable risk group HL patients treated with either chemotherapy alone or with CMT. Age distribution of our patients was consistent with published data.^28^ Both groups (chemo alone and CMT) were balanced. The majority of patients had ABVD regimen. Dose range of involved field radiotherapy was 20 to 36 Gray. Patients treated with CMT showed significant progression free and overall survival benefit, p=0.01 and p=0.03, respectively. Our results are consistent with previous studies revealing survival benefit with CMT.^29^ Interestingly with a median follow-up of more than 50 months, Overall survival was 100% at 5 years in CMT. It means there were no deaths due to disease or toxicity which is very encouraging to use CMT. This is consistent with persisting evidence that CMT is effective and less toxic than higher doses of chemotherapy and radiation.^5^ Furthermore, this difference was maintained at 10 years as well with projected PFS and OS better with CMT. As far as toxicities are concerned, two patients in CMT and one in chemo alone group had pulmonary toxicity. Hospital admissions were four with CMT and three with chemo group. Again the difference between two groups does not seem to be very significant. More relapses were encountered in chemotherapy alone group. One second malignancy was noticed in CMT group. Because of the small number of patients, the significance of these results cannot be determined statistically.

Based on above results, for early stage–favorable Hodgkin lymphoma, CMT resulted in better outcomes compared to chemotherapy alone. However, each patient should be dealt with on individual basis. For example, in a young female with favorable disease (who may require radiotherapy to chest as a part of CMT) chemotherapy alone may be better option-keeping in mind risk of breast cancer with radiotherapy. Similarly, radiotherapy should be avoided in pregnant female where chemotherapy alone will be favored. ABVD regimen is relatively safe in pregnancy; in particular during 2^nd^ and 3^rd^ trimester.^30^

## CONCLUSIONS

Our analysis is consistent with findings from clinical trials showing that combined modality treatment is safe and effective for favorable risk HL. However, in certain cases such as young females or during pregnancy radiation may be avoided and patient could be treated with chemotherapy alone to decrease the risk of breast cancer in long run. Best way of finding the most optimal treatment for early stage Hodgkin lymphoma will be to design more prospective studies with larger number of patients and longer follow ups.
